# Incorporating ‘Green Podiatry’ into your clinic, and into your life

**DOI:** 10.1186/s13047-022-00591-y

**Published:** 2022-12-09

**Authors:** Angela Margaret Evans AM

**Affiliations:** Discipline of Podiatry, School of Allied Health, Human Services and Sport, La Trobe University, Melbourne, 3086 Australia

**Keywords:** Climate change, Footprint, Healthcare, Carbon, Podiatry, Emissions, Health, Green

## Abstract

**Background:**

This commentary outlines practical ways of positively incorporating green podiatry, foot health, physical activity benefits, and relevance to climate change into the clinical setting as Conference of Parties (COP27) approaches. Recent reports from the Intergovernmental Panel on Climate Change, the World Economic Forum, and undergraduate curricula concerns, are presented.

**Main body:**

Climate change is irrefutable, and as health professionals, podiatrists can discuss the benefits and principles of green podiatry with patients of all ages in their clinics, appreciating that people are increasingly worried about the climate crisis.

Feet as fundamental for independent, healthy, and carbon–neutral active transport, needs to become a key message. The three pillars for green podiatry are exercise, evidence, and the everyday changes that all podiatrists can make. Likewise, podiatrists can encourage their patients, and in doing so, join with community leadership, alongside other allied health and medical peers.

**Conclusion:**

Podiatrists have a shared responsibility to work and live as ‘green’ as possible, and to share this message with patients. Reducing waste, physically and in the form of unnecessary treatment, and supporting a review of supply chains, are important aspects of reducing health care emissions.

Promoting feet as carbon–neutral transport, and physical activity as evidence based and health enhancing, are a sound contribution to twenty-first century public health. Podiatry has a great opportunity for positive legacy.

**Supplementary Information:**

The online version contains supplementary material available at 10.1186/s13047-022-00591-y.

## Background

The concept and initiative of ‘Green Podiatry’ was introduced previously [1], followed by a Sustainability panel review prior to COP26 [2]. This commentary is linked to two previous commentaries and outlines new research from the Intergovernmental Panel on Climate Change (IPCC),^1^ The World Economic Forum,^2^ and academics [3, 4], to outline practical ways of positively incorporating CC into the clinical setting, as COP27 approaches.^3^ We all need to act to avert the existential threat before us. Podiatrists should be well versed to reduce carbon *footprints!*

Three key principles to consider are: Climate change is irrefutable; Talk with Patients about ‘Green Podiatry’; We must listen to important groups.

### Climate change is irrefutable

*‘It is unequivocal that human influence has warmed the atmosphere, ocean and land. Widespread and rapid changes in the atmosphere, ocean, cryosphere and biosphere have occurred’*: the latest IPPC report summarises the impact of climate change from thousands of scientific papers. This report covers many topics, including: water systems, food systems, Indigenous knowledge, oceans, cities, health, poverty and inequality. The 3000 pages present three salient points [1]  (Supplementary files 1, 2):

1. Climate change is already harming people’s health
2. More action is needed to protect health
3. Climate solutions benefit both health and the economy [5].

### Talk with patients about ‘Green Podiatry’

Health professionals are in a unique and trusted position to speak with patients about the impact of CC on health, and it is timely. Covid-19 made 47% of Australians more concerned about CC [6], and three times more worried about CC than about Covid-19 [6]. Women, young adults, the affluent, and ages 35 to 54 years, showed most concern about CC. The middle-aged may worry because they are parents, and naturally want a secure future for children. Concern among younger adults (18 to 34 years) is understandable, given the existential crisis before them.^4^

As individual citizens we share lifestyle challenges, providing a great opportunity for podiatrists to ‘lead by example’ on the key factors that both mitigate CC, and improve health (Supplementary file 3).

### We must listen to important groups

a *Students and young people*

The ‘Foundations for Tomorrow’ survey (World Economic Forum [2]), had 10,000 responses from Australians aged under 30 years. Results were:

- 93% saying the government was not doing enough to address CC
- 75% said they would vote for political leaders taking bold action on CC
- 11% felt their vote mattered

In 2021, Australian teenagers successfully sued the Minister for Environment^5^ regarding the government’s duty of care to future generations on CC.^6^ This decision was later overturned, with the Minister abrogating responsibility via Federal Court appeal. Six months later, the government lost the federal election, with a ground swell of ‘green’ and ‘teal’ votes.^7^ CC was a decisive election issue^8^.

Globally, ‘Fridays for Future’ *(‘Greta effect’*), continue as a youth CC initiative [7]. Children and young people are worried, and addressing this is key to managing eco-anxiety (https://www.climatecouncil.org.au/guide-parents-managing-eco-anxiety-your-kids/ [8–10]). Children´s rights, mental health, and CC are future challenges for children now, and in subsequent generations [11].

Podiatry students in the UK (University of Southampton) have investigated and reported on a sustainability agenda. Their work was presented at the Royal College of Podiatry (RCPod) conference, July 2022. Results of an online member survey (*n* = 75), found:

- 93.3% thought sustainability within Podiatry was important
- 73% thought the topic was important for business
- Respondents identified both barriers and opportunities enhance sustainability.^9^

b *Indigenous people*

Comprising less than 5% of the world's population, *indigenous people* protect 80% of global biodiversity.^10^ Emerging in 2017, and pending referendum, the ‘Uluru Statement from the Heart’ represents a historic consensus of indigenous leaders in seeking constitutional change to recognise First Australians, and is an invitation from the Aboriginal and Torres Strait Islander people to,

“Walk with us in a movement of the Australian people for a better future”.^11^

### Accelerating climate action: the role of health professionals and systems

“The climate emergency is a multidisciplinary, multisectoral, crisis that transcends professional and organisational barriers. Health professionals can help bring sharp focus to the urgent reforms required from individuals, organisations, and governments” [3].

I share the view that sustainability in practice, begins with embedding CC in university curriculums [4]. The NHS has ambitious targets to reach net zero over the next two decades. Clinicians will require knowledge and support to achieve this goal, as part of the NHS responsibility for achieving climate targets [12, 13].

Climate action needs promotion in public health to include the large emission areas, eg transport, energy, food, agriculture, housing; to reduce air pollution, increase physical activity, improve diets [14, 15].

A fresh focus on ‘big picture health’,^12^ must balance/lessen the management of illness. Medical ‘prescribing’ can prioritise ‘green health’, to encourage time in the natural world as therapeutic, as occurs in Canada [16], Scotland [17], and China [18].

### Green Podiatry – Pillars for Practice

Green Podiatry is founded on: 1) exercise, 2) evidence, and 3) everyday actions^13^ (Table 1). Calculation of your carbon footprint, at work and at home, is illuminating, and provides a baseline for targeting change eg,

- walk to the shops rather than drive
- use public transport
- walk/cycle to work (even once/week), and encourage patients similarly (https://www.footprintnetwork.org/).

**Table 1:** The three pillars of Green Podiatry for clinicians. Assess carbon calculations – work (UK) https://www.gpcarbon.org/#/; and at home (Australia): https://www.wwf.org.au/get-involved/change-the-way-you-live/ecological-footprint-calculator#gs.u5agd1

**Green Podiatry Pillars**		
**Exercise**	**Evidence**	**Everyday**
**Feet as C-neutral transport** Walk, ride, swim, run, dance ⦁ Good for people ⦁ Good for the planet ⦁ Evidence-based	**Stop treatment which is not evidence-based** eg Bespoke foot orthoses for paediatric flat feet	**Supply chains are the major healthcare emission** ⦁ Use less of everything ⦁ Reuse, repair ⦁ Recycle, reform (circular economy) ⦁ Buy from local sources ⦁ Adopt active travel for work (even once/week)
Feet for exercise should be the primary podiatry focus for every patient Use wearable technology to measure physical activity General advice ⦁ Screen time ⦁ Diet ⦁ Sleep ⦁ Exercise ‘dosage’/age https://www.who.int/news-room/fact-sheets/detail/physical-activity	Use ‘choose wisely’ questions with patients: 1. Do I really need this test, treatment or procedure? 2. What are the risks? 3. Are there simpler, safer options? 4. What happens if I don't do anything? 5. What are the costs? https://www.choosingwisely.org.au/resources/consumers-and-carers/5questions Attend online webinars, reduce your travel emissions, especially long flights	Energy ⦁ electrify with renewables ⦁ e-records, telehealth Minimise single use items Collection bins: ⦁ Paper ⦁ Plastics ⦁ e-waste ⦁ Footwear to recycle

Such simple changes are good for health, good for our planet, and raise awareness of both [19].

1. *Exercise*

Podiatrists need to focus on foot health for carbon–neutral transport. Physical activity is easily measured with wearable technology (phones, watches, fit-bits) and ‘dosed’, eg adults: 300 min moderate-vigorous physical activity/week, and more if sedentary^14^ [19]. This is essential primary HC for podiatrists to champion, and a great antidote for non-communicable diseases (eg diabetes, arthritis, obesity, depression, cancer, heart disease). Exercise may allay ‘eco-anxiety’ in children, with cycling fostering children’s well-being, and independent transport [20].

A ‘green shoe list’ [www.evidenceessentials.com; https://angelaevanspodiatrists.com.au/green-shoes/ accessed 4 Jul 2022] acknowledges that exercise involves feet, and footwear. Podiatry needs transparency from footwear manufacturers, to avert ‘green-washing’ for commercial gain^15^. Aware manufacturers increasingly use natural materials, including raffia palm and banana skin fibres [21]. Footwear can be repaired and recycled (access local footwear recycling^16^), and dedicated companies offer ‘take back’ programmes,^17^ and even shoes for lease^18^.

The Green Foot Orthoses Project (GFOP) is a new initiative,^19^ and foot orthoses use must be supported by diagnosis, and evidence. Repairing orthoses further extends product life.

2. *Evidence*

We can encourage ‘health’, over ‘healthcare’. In the lower limb, knee arthroscopy [22], and customised foot orthoses for paediatric flat feet [23], are interventions no longer evidence-based, and clinicians should stop using them. HC, needs to focus on evidence based care, and dispense with unnecessary treatments, imaging, tests [24, 25]. We can engage patients in ‘Wiser Healthcare’ to avert overdiagnosis [26], and excessive HC.^20^ Podiatrists are in prime position to promote healthy feet for physical activity which aids health [27], is evidence based [19]and provides carbon neutral transport [14].

3. *Everyday*

The first commentary outlined changes for podiatrists to lessen GHG emissions [1]. Fossil fuels comprise 98% of plastics, and approximately 40% of plastics are single-use.^21^ HC is a large user, especially hospitals,^22^ where use of PPE and single use items creates enormous waste. Pegna and McNally [28] have suggested a pause:

‘We are constantly told that for ‘infection control reasons’ we must wear and use single use items. But where is the evidence for this?

Is there evidence that single use items are always safer than reusable ones?

Is there evidence that disposable drapes are better than washable for infection prevention?’

The NHS, and CAHA support the Global Green and Healthy Hospital network to reduce environmental impacts of workplaces.^23^ Supply chains cause most emissions (approximately 70%), and RCPod, APodA could partner with suppliers, footwear manufacturers, waste hubs (eg Treadlightly^24^), to access ‘green’ supplies, and circular economy waste cycles.

## Conclusion

Ultimately, we each share the responsibility to work and live as ‘green’ as we can. What we do as podiatrists – reduce waste, reduce unnecessary treatment, promote and live ‘green podiatry’—and as citizens – buy local, buy less, choose renewable energy, vote thoughtfully, divest fossil fuel investments, manage waste – is important for every aspect of health.

Two suggestions for podiatrists are to: 1) adopt the three pillars of Green Podiatry, viz., exercise, evidence, and everyday practices; 2) promote feet as carbon–neutral transport, and physical activity as evidence based and health enhancing. Podiatry has a great opportunity for positive legacy.

## Supplementary Information

**Additional file 1.** Intergovernmental Panel on Climate Change, summary of 6^th^ report, 2021.

**Additional file 2.** Impact summary of climate change from review of thousands of scientific papers – IPCC, 2021.

**Additional file 3.** Green podiatry health education conversation outline.

## Data Availability

N/A.

## Abbreviations

CC: Climate change
HC: Healthcare
COP26: Conference of Parties, Glasgow, November 2021
COP27: Conference of Parties, Egypt, November 2022
IPPC: Intergovernmental Panel on Climate Change
GHG: Green-house gases
LMIC: Low-and-middle-income countries
UK: United Kingdom
RCPS (Glasg): Royal College of Physicians and Surgeons (Glasgow)
GFOP: Green Foot Orthoses Project
PPE: Personal protective equipment
RCPod: Royal College of Podiatry
APodA: Australian Podiatry Association
ESG: Environment Social and Governance (policy)
CO^2^: Carbon dioxide
NH^4^: Methane
WHO: World Health Organisation
UN: United Nations
DEA: Doctors for the Environment Australia
CAHA: Climate and Health Alliance
NHS: National Health Service

## Footnotes

1 IPCC Report 2021: https://www.ipcc.ch/report/ar6/wg2/ Accessed 20 October 2022.

2 Foundations for Tomorrow Report: https://www.foundationsfortomorrow.org/the-report Accessed 20 October 2022.

3 UN Climate Change Conference 2022 (COP 27): https://sdg.iisd.org/events/2021-un-climate-change-conference-unfccc-cop-27/ Accessed 20 October 2022.

4 The Conversation, Fri 6 Aug 2021, https://www.abc.net.au/news/2021-08-06/australians-three-times-more-worried-about-climate-change-covid/100354008 Accessed 20 October 2022.

5 Federal Court of Australia: https://www.judgments.fedcourt.gov.au/judgments/Judgments/fca/single/2021/2021fca0560 Accessed 20 October 2022.

6 The Guardian, 21 September, 2021: https://www.theguardian.com/environment/2021/sep/01/young-australians-screaming-for-climate-action-but-dont-trust-leaders-to-make-change-survey-suggests Accessed 20 October 2022.

7 Green and teal candidates – independent of either major (Labour/Liberal) parties with strong policy for CC.

8 The Guardian, 23 May, 2022: https://www.theguardian.com/australia-news/2022/may/23/teal-independents-who-are-they-how-did-they-upend-australia-election Accessed 20 October 2022.

9 RCPod Abstract, 2022.

10 National Geographic Society: https://www.nationalgeographic.com/environment/article/can-indigenous-land-stewardship-protect-biodiversity- Accessed 20 October 2022.

11 The Uluru statement from the Heart: https://fromtheheart.com.au/ Accessed 20 October 2022.

12 https://www.podiatry.org.au/documents/item/2272. Accessed 31 May 2022.

13 President’s webinar, Royal College of Physicians and Surgeons of Glasgow, 7 June, 2022 (members: https://learning.rcpsg.ac.uk/mod/scorm/player.php?a=1273&currentorg=articulate_rise&scoid=2624&sesskey=CaIQ1gTvVz&display=popup&mode=normal).

14 WHO physical activity fact sheets https://www.who.int/news-room/fact-sheets/detail/physical-activity Accessed 20 October 2022.

15 Australian Institute of Company Directors (AICD) greenwashing https://www.aicd.com.au/risk-management/framework/climate/whats-on-the-watchlist.html Accessed 20 October 2022.

16 Treadlightly footwear recycling https://treadlightly.asga.com.au/ Accessed 20 October 2022.

17 Etiko fair trade footwear and clothing https://etiko.com.au/ Accessed 20 October 2022.

18 On footwear https://www.on-running.com/en-au/cyclon Accessed 20 October 2022.

19 University of New South Wales – Sustainable Materials Research and Technology (SMaRT) rehttps://www.smart.unsw.edu.au/search?keywords=podiatry Accessed 20 October 2022.

20 Choose wisely https://www.wiserhealthcare.org.au/questions-to-ask-a-health-professional/ Accessed 20 October 2022.

21 World Wildlife Fund (WWF) https://www.wwf.org.au/news/blogs/10-worst-single-use-plastics-and-eco-friendly-alternatives#gs.u5l02t Accessed 20 October 2022.

22 Climate and Health Alliance (CAHA) https://www.caha.org.au/reducing_sup_in_health Accessed 20 October 2022.

23 Global Green and Healthy Hospitals (GGHH) https://greenhospitals.org/ Accessed 20 October 2022.

24 Treadlightly footwear recycling https://treadlightly.asga.com.au/ Accessed 20 October 2022.
