# Supplementary material for: Incorporating ‘Green Podiatry’ into your clinic, and into your life
Source: J Foot Ankle Res. 2022 Dec 9;15:87. doi: 10.1186/s13047-022-00591-y (PMC9733335; doi:10.1186/s13047-022-00591-y)
Supplement: Supplementary file 1 — Additional file 1. Intergovernmental Panel on Climate Change, summary of 6th report, 2021. [file 13047_2022_591_MOESM1_ESM.docx]

Commentary: Incorporating ‘Green Podiatry’ into your clinic, and into your life

Supplementary file 1

**Intergovernmental Panel on Climate Change, summary of 6^th^ report, 2021**

- Each of the last four decades has been warmer than any decade preceding it, since 1850.
- Global surface temperature was 1.09°C higher in 2011–2020 than 1850–1900
- GHGs were the main driver of tropospheric warming since 1979
- Global mean sea level has risen faster since 1900 than over any preceding century in at least the last 3000 years, also more acidic.
- Human-induced climate change is already affecting many weather and climate extremes in every region across the globe.
- Evidence of observed changes: heatwaves, heavy precipitation, droughts, tropical cyclones, has strengthened
- In 2011–2020, annual average Arctic sea ice area reached lowest level since 1850.
- Marine heatwaves have almost doubled in frequency since the 1980s
- Climate change now affects every inhabited region across the globe with human influence contributing to many observed changes in weather and climate extremes
- Every additional 0.5°C of global warming causes clear increases in the intensity and frequency of hot extremes, viz. heatwaves, heavy precipitation, and droughts.
- Physical science: limiting human-induced global warming requires limiting CO^2^ emissions, to reach net zero CO^2^ emissions. Further, rapid and sustained reductions in CH^4^ emissions would also limit the warming effect and improve air quality.
- Every tonne of CO^2^ emissions adds to global warming.
- There is a near-linear relationship between cumulative anthropogenic CO^2^ emissions and the global warming, ie 1000 Gt CO^2^ of cumulative CO^2^ emissions equates to a 0.45°C increase in global surface temperature (0.27°C to 0.63°C).

Climate Change 2021. The Physical Science Basis Working Group, Contribution to the Sixth Assessment Report of the Intergovernmental Panel on Climate Change, Masson-Delmotte, V., P. Zhai, A. Pirani, S. L. Connors, C. Péan, S. Berger, N. Caud, Y. Chen, L. Goldfarb, M. I. Gomis, M. Huang, K. Leitzell, E. Lonnoy, J.B.R. Matthews, T. K. Maycock, T. Waterfield, O. Yelekçi, R. Yu and B. Zhou (eds.)]. Cambridge University Press, ISBN 978-92-9169-158-6; [www.ipcc.ch](http://www.ipcc.ch)
