# Supplementary material for: Incorporating ‘Green Podiatry’ into your clinic, and into your life
Source: J Foot Ankle Res. 2022 Dec 9;15:87. doi: 10.1186/s13047-022-00591-y (PMC9733335; doi:10.1186/s13047-022-00591-y)
Supplement: Supplementary file 2 — Additional file 2. Impact summary of climate change from review of thousands of scientific papers – IPCC, 2021. [file 13047_2022_591_MOESM2_ESM.docx]

Commentary: Incorporating ‘Green Podiatry’ into your clinic, and into your life

Supplementary file 2

**Impact summary of climate change from review of thousands of scientific papers – IPCC, 2021**

The crucial geographical regions of Pacific Islands, and other low-and-middle-income countries (LMIC), will experience the greatest CC effects.

1. Climate change is already harming people’s health

- illness, injury, death from extreme weather (floods, bushfires, heatwaves)
- increased spread of mosquito and tick-borne disease
- more cardiovascular disease from extreme heat
- respiratory diseases from air pollution
- food insecurity
- increased risks for mental health and wellbeing
- effects on livelihoods, migration, conflict.

The health impacts of CC are real and worsening. Disadvantaged people are most affected, increasing health inequity. Rapid cuts to greenhouse gas (GHG) emissions and adaptation strategies can reduce future health risks.

1. More action is needed to protect our health

The solutions to reduce the health risks from CC are available, but we need more action to anticipate the health impacts, identify vulnerable populations, and improve health protection. Reducing socio-economic inequalities is a large component in managing climate-related health risks.

1. Climate solutions benefit both health and the economy

The benefits to health far exceed the costs of climate action, which is good for health. Strategies to reduce greenhouse gas emissions and strengthen resilience have significant benefits for health and wellbeing, eg

- cleaner air, soil, water
- improved mental health
- more active and resilient communities
- healthier diets.
