# Supplementary material for: Incorporating ‘Green Podiatry’ into your clinic, and into your life
Source: J Foot Ankle Res. 2022 Dec 9;15:87. doi: 10.1186/s13047-022-00591-y (PMC9733335; doi:10.1186/s13047-022-00591-y)
Supplement: Supplementary file 3 — Additional file 3. Green podiatry health education conversation outline. [file 13047_2022_591_MOESM3_ESM.docx]

Commentary: Incorporating ‘Green Podiatry’ into your clinic, and into your life

Supplementary file 3

**Green podiatry health education conversation outline**

- Healthy feet are important for your health and mobility
- The right amount of regular exercise is associated with better health, including:
  - cardiovascular fitness, physical strength, better sleep and mental health
  - exercise and a good diet help to maintain weight, blood pressure, bone health
  - exercise needs to be ‘dosed’ according to age, sedentary time, level of vigour ^20^.
- Good feet underpin most exercise, eg walking, swimming, cycling, running, rowing
- Healthy feet provide independent transport, which is carbon neutral, and keeps our air cleaner to breathe
- Use public transport whenever you can, and walk/cycle locally rather than drive
- We need a healthy planet, to have healthy people, eg clean air, clean water
- Together, we can all act positively to lessen climate change
- You can make your shoes last longer – repairs can extend shoe life
- Did you know that footwear may be recycled to avoid landfill? (provide recycling locations, consider a collection bin in your clinic)
- Did you know that some footwear has gone ‘green’?
- As podiatrists we focus on feet for health and exercise, and reducing our carbon footprints as much as possible
